# Comparison between contrast-guided and pressure-guided ablation using the novel pressure visualization tool for cryoballoon pulmonary vein isolation

**DOI:** 10.1007/s00380-025-02574-y

**Published:** 2025-07-08

**Authors:** Philipp Bengel, Helge Haarmann, Eva Rasenack, Nibras Soubh, Simon Schlögl, Gerd Hasenfuß, Markus Zabel, Leonard Bergau

**Affiliations:** 1 Department of Cardiology and Pneumology, Heart Center, University Medical Center Göttingen, Robert-Koch-Str. 40, 37075 Göttingen, Germany; 2 German Center of Cardiovascular Research (DZHK), Partner Site Göttingen, Göttingen, Germany; 3 Department of Cardiology, Justus-Liebig University of Giessen, Giessen, Germany

**Keywords:** Cryoballoon, Occlusion pressure, POLARx, Atrial fibrillation, Pulmonary vein isolation

## Abstract

During cryoballon pulmonary vein isolation (PVI) complete occlusion of the pulmonary vein ostia during the freeze cycles is mandatory. Typically, PV occlusion is assessed by contrast injection under fluoroscopy. Using an update for the Cryo Console it is possible to directly visualize occlusion pressure as an indicator of complete PV occlusion during cryoballoon procedures. In this study, we compared PV pressure monitoring during cryoballoon PVI to a conventional approach regarding procedural outcomes. We retrospectively analysed the procedural data of 50 patients (25 patients with pressure-guided PVI and 25 patients with contrast-guided PVI) treated with cryoballoon PVI in our centre. Complete PV occlusion in the pressure-guided group was defined as an abrupt change in the pressure waveform with a loss of the a-wave after advancing the cryoballoon to the PV ostium. We observed comparable results regarding procedural time, left atrial dwell time or fluoroscopy time when comparing the pressure guided to our conventional approach. Moreover, there were no differences regarding acute procedural effectivity or freeze cycle characteristics. As expected, a significant reduction of contrast use was achieved in the pressure measurement group (10.4 vs. 25.5 ml, *p* < 0.0001). Monitoring complete PV occlusion by visualizing the occlusion pressure is feasible. Acute procedural outcome was comparable to our standard approach using contrast injection to verify complete PV occlusion. Most importantly, a significant reduction in contrast use could be achieved which has to be confirmed in larger patient cohorts.

## Introduction

Pulmonary vein isolation (PVI) using radiofrequency ablation (RFA) or cryoballoon (CB) ablation has emerged as standard treatment for symptomatic paroxysmal and persistent atrial fibrillation (AF) [1, 2]. Both techniques are comparable in terms of long-term durability of PVI and freedom of AF in patients with paroxysmal AF [3–5]. Moreover, in these patients, CB-ablation as first-line therapy is superior compared to a conventional antiarrhythmic drug therapy [6, 7]. However, compared to RFA, the CB-PVI has several advantages, including a shorter procedure duration, a higher degree of reproducibility, and a shorter learning curve [8, 9].

For CB-PVI, complete occlusion of the pulmonary vein (PV) ostium is crucial to achieve an effective and durable lesion [10]. PV occlusion is usually verified by contrast agent injection. However, the use of contrast agent might be limited due to the patients’ condition such as impaired renal function or contrast agent intolerance and causes additional costs. Pressure measurement at the distal tip of the CB-catheter to ensure complete occlusion has been discussed as alternative strategy, but no dedicated solution was yet available [11, 12].

The POLARx® (Boston Scientific, USA) system is a CB-ablation system introduced in Europe in 2020. Clinical studies proved equality between the novel system and established systems [13–15]. An update was recently introduced which allows visualization of the pressure at the tip of the catheter at the console.

This study aimed to evaluate the feasibility as well as cryoablation characteristics and acute efficacy of the novel occlusion pressure visualization (OPV) tool of the SMARTFREEZE® console to verify sufficient PV occlusion in comparison to a conventional contrast-guided approach during CB-PVI.

## Materials and methods

### Study design and patient selection

We retrospectively analysed procedural data from 50 patients who underwent de novo PVI using the POLARx cryoballoon. The OPV was used for CB-PVI in our centre as soon as it became available in November 2022. Patients were not randomized, and procedures were performed by the same three operators only. Patients were ablated with the OPV tool from the moment it became available, the control group consisted of the last 25 patients that were ablation with CB before. Patients showing a left common PV ostium upon a preprocedural cardiac CT scans were excluded from analysis. The study was approved by the local ethics committee and conducted in accordance with the Declaration of Helsinki. Informed consent was obtained from all individual prior the procedure.

### Ablation procedure

Procedural management was similar between the two groups until advancing the cryoballoon catheter to the LA. Procedures were performed under deep sedation using midazolam, fentanyl and propofol. Anticoagulation was paused on the morning of the procedure in patients treated with direct oral anticoagulants (DOACs), anticoagulation with Vitamin K antagonists (VKA) was continued targeting an INR of 2–2.5 for the day of the procedure. Two left and one right femoral vein punctures were performed and 2 ×  7 F (left) and 1 ×  8 F (right) short sheaths were inserted. Prior to transseptal puncture (TSP), a diagnostic decapolar catheter was introduced via the left femoral vein and positioned within the coronary sinus (6F; Dynamic Tip, Boston Scientific, MA, USA). Patients in atrial fibrillation at the beginning of the procedure underwent electrical cardioversion right after transseptal access. A total of 5000 IE of heparin was administered followed by a second heparin bolus after TSP (to achieve 100 IE/kg body weight for both boluses). Single TSP was performed under fluoroscopic guidance via a modified Brockenbrough technique using a BRK1-needle and an SL1-8.5F transseptal sheath (St. Jude Medical, Inc.) followed by selective angiography of all PVs. Then the SL1-sheath was exchanged for the 15.9F POLARSHEATH over the wire. The sheath was continuously flushed with heparinized saline (20 mL/h). During the procedure, heparin boluses were administered targeting an activated clotting time of > 300 s.

Then, the 28 mm POLARx CB [POLARx short tip (ST) Boston Scientific] was advanced to the LA over a 20 mm spiral mapping catheter (10-polar, PolarMap, Boston Scientific) as a guidewire. For ablation the PolarMap was introduced into the respective PV and the cryoballoon (CB) was inflated proximal to the PV ostium. After inflation, the CB was positioned at the ostium. Complete occlusion of the PV was verified with either contrast dye injections or pressure curve observation as described in detail below (“Conventional group” and “OPV group” sections). The PolarMap was positioned at a proximal position to achieve live PV recordings and measurements of time to isolation (TTI).

The minimal CB temperature cut-off was set to − 70 °C, during ablation of the right PVs the phrenic nerve was continuously paced. Capture was monitored by tactile feedback of diaphragmatic contraction in conjunction with the digital movement sensor (DMS) provided by the POLARx system. Ablation was immediately stopped if weakening or loss of diaphragmatic contraction was noticed.

The standard duration of a single freeze was 180 s. If TTI was > 60 s, the freeze cycle was prolonged to the operators’ discretion to 210 s or 240 s with a minimum of 120 s in addition to TTI [16]. The PVs were treated in a clockwise sequence [left superior pulmonary vein (LSPV), left inferior pulmonary vein (LIPV), right inferior pulmonary vein (RIPV), right superior pulmonary vein (RSPV)]. After 70 s of freezing a pull down manoeuvre was performed for LIPV and RIPV [13, 17]. Isolation of all veins was proved by entrance and exit block.

#### Conventional group

**Fig. 1:**
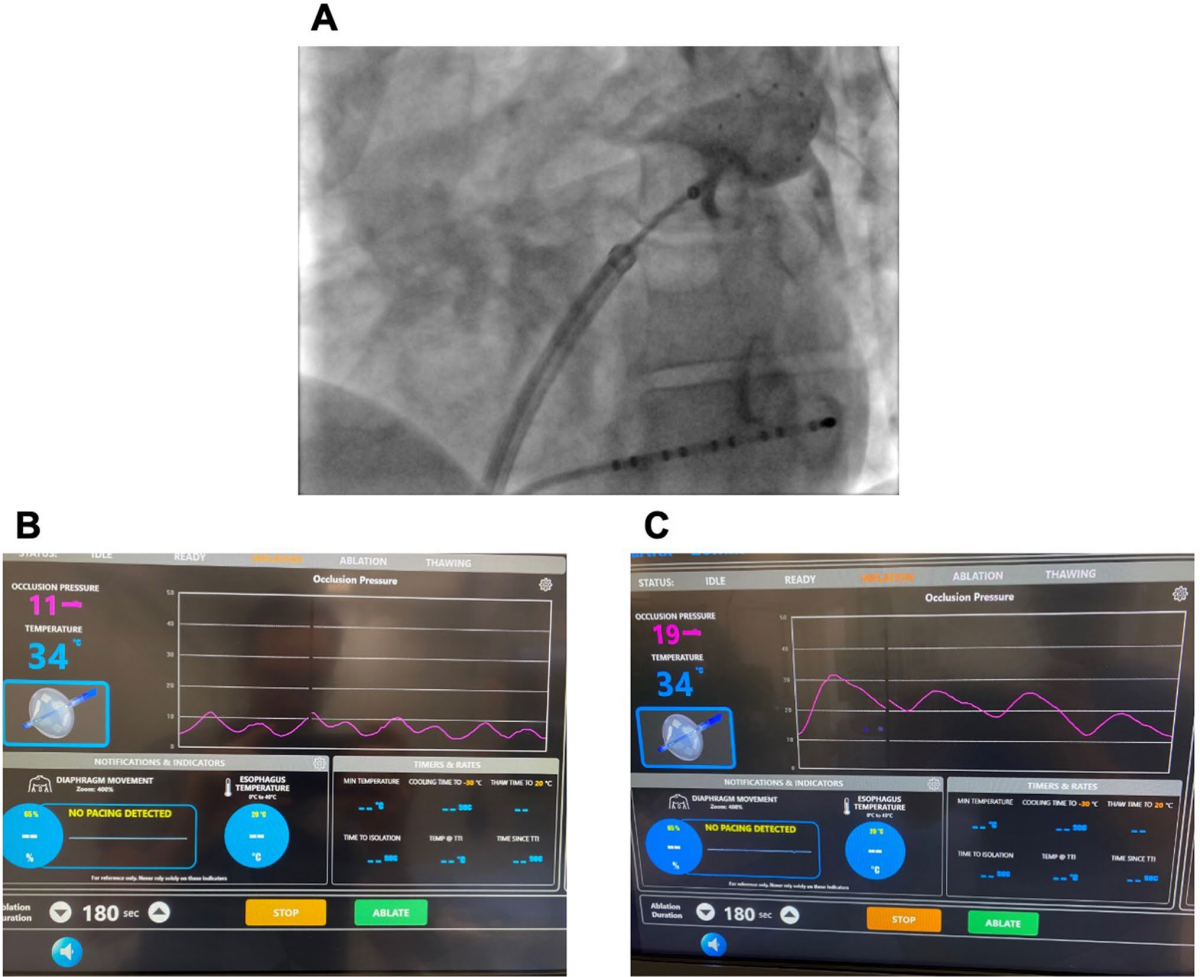
Two different approaches to verify complete PV occlusion. **A** Fluoroscopic example of the cryoballoon advanced to the LSPV, contrast is caged within the PV after injection displaying a complete occlusion. **B** Pressure waveform recorded from the tip of the cryoballoon catheter showing an atrial pressure curve containing an a-wave and a v-wave. **C** Pressure waveform recorded from the tip of the cryoballoon catheter after complete occlusion of the PV, showing a complete loss of the a-wave and a larger v-wave. This pressure corresponds to the pulmonary artery pressure

In the contrast-guided (CG) group, occlusion of the PVs was verified by contrast injection after advancing the inflated balloon to the PV ostium and before starting the freeze (Fig. 1A). Ablation was started only when a stable occlusion was verified 3 s after initial contrast injection. In the case of outflow of contrast the balloon was repositioned and contrast was injected again until complete occlusion was achieved before starting ablation.

#### OPV-group

Confirmation of the PV Occlusion in the pressure-guided group was ascertained by pressure visualization via a single pressure transduce connected to the inner lumen of the CB. The pressure waveform was displayed on the SMARTFREEZE® console as shown in Fig. 1 (Fig. 1B, C). After balloon advancement, the transition of the pressure waveform from an LA pressure (Fig. 1B) to a wedged PV tracing (Fig. 1C) equivalent to the pulmonary artery pressure waveform. The switch was considered as complete PV occlusion. Total PV occlusion resulted in a loss of the A (atrial) wave with an increase in slope and amplitude of the V (ventricular) wave. Fluoroscopy was used to confirm an antral position of the CB.

### Postprocedural care

After removal of the sheaths a figure-of-eight suture, and a pressure bandage were used to prevent femoral bleeding. After 6 h, the pressure bandage was removed; the figure-of-eight suture was removed the next day. Directly after the procedure, as well as 4 h later pericardial effusion was ruled out by transthoracic echocardiography. Anticoagulation with DOACs was restarted in the evening of the procedure after pericardial effusion was ruled out. Anticoagulation with VKA was continued targeting an INR of 2–3. Proton-pump inhibitors were started for 4 weeks after the procedure. Anticoagulation was continued at least 3 months and afterwards continuation was decided based on individual stroke risk assessed by CHA_2_DS_2_-VASc-Score.

### Data collection

Data on patients'characteristics, medication, symptoms, and complications were compiled from patients'records and discharge letters. Procedural parameters were taken from ablation protocols and procedure-related documents. Data were collected retrospectively.

### Statistical analysis

Differences of metric variables between the two groups were analysed using a *t* test if the data were normally distributed, and with the Wilcoxon-Mann–Whitney *U* test otherwise. Differences between categorical variables were evaluated using the Fisher’s exact test. All *p* values are two sided and a *p* value < 0.05 was considered significant. All calculations were performed with the statistical analysis software, GraphPad Prism 9 for MacOS (GraphPad Software, Version 9.5.1, Boston, Massachusetts USA, www.graphpad.com).

## Results

### Patient characteristics

A total of 50 patients were included in the analysis (25 patients per group). Patient baseline characteristics are shown in Table 1. No imbalances were detected between the groups.

**Table 1:** Patient baseline characteristics

Variable	Contrast guided	Pressure guided	*p* value
Age (years)	67.0 ± 6.9	64.8 ± 11.5	0.424
Body mass index	31.5 ± 6.4	30.1 ± 6.1	0.436
Female (%)	11 (44)	12 (48)	> 0.999
Persistent AF (%)	18 (72)	13 (52)	0.244
EHRA-Score	2.9 ± 0.6	2.8 ± 0.4	0.771
CHA_2_DS_2_-VASc-Score	3.2 ± 1.4	2.5 ± 1.2	0.082
Ejection fraction (%)	47.4 ± 10.9	51.7 ± 10.6	0.168
LAVI (ml/m^2^)	40.1 ± 13.0	36.1 ± 9.2	0.259
Coronary artery disease (%)	3 (12)	7 (28)	0.289
COPD (%)	4 (16)	2 (8)	0.667
Arterial hypertension (%)	20 (80)	18 (72)	0.742
Obstructive sleep apnoea (%)	6 (24)	1 (4)	0.098
Diabetes mellitus (%)	7 (28)	4 (16)	0.496
Prior cardioversion (%)	20 (80)	15 (60)	0.217
Beta-blocker (%)	23 (92)	21 (84)	0.667
DOAC (%)	25 (100)	21 (84)	0.110
VKA (%)	0 (0)	3 (12)	0.235
Antiarrhythmics (%)	11 (44)	9 (36)	0.773
Suspected AIC (%)	7 (28)	3 (12)	0.289
Device (%)	5 (20)	3 (12)	0.702

AF, atrial fibrillation; EHRA, European Heart Rhythm Association; LAVI, left atrial volume index; COPD, chronic obstructive pulmonary disease; DOAC, direct oral anticoagulants; VKA, vitamin K antagonist; AIC, arrhythmia induced cardiomyopathy

### Procedural characteristics

**Fig. 2:**
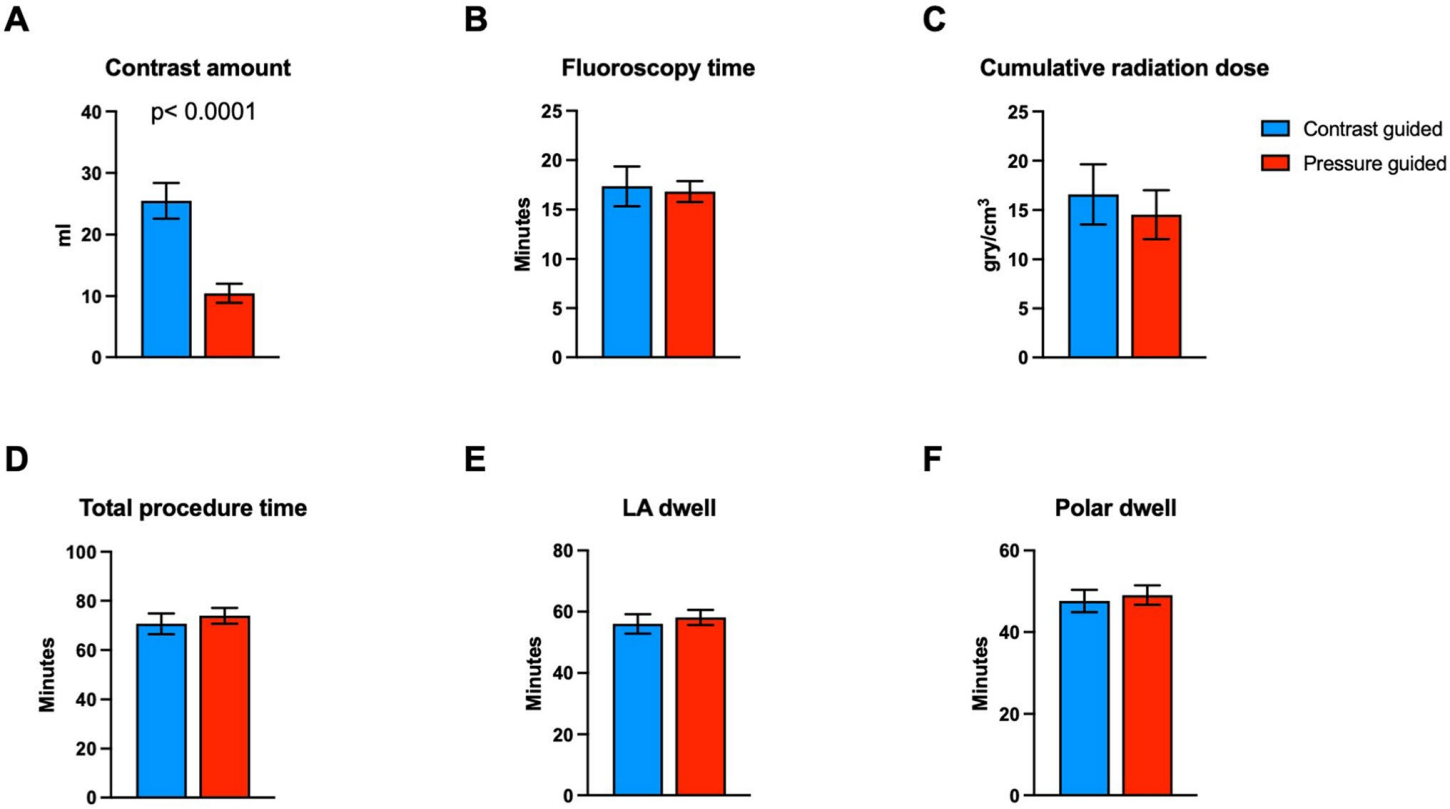
Comparison of procedural characteristics between the contrast-guided and the pressure-guided group. **A** Significantly, less contrast agent was necessary in the pressure-guided group. **B**–**F** There were no differences observed regrading fluoroscopy time, cumulative radiation dose, total procedure time, LA dwell time or LA dwell time of the POLARSHEATH between the procedure guided with contrast or guided by pressure waveform analysis

The use of pressure measurement in the OPV group was associated with a significant reduction of contrast agent use (Fig. 2A, p < 0.0001) as compared to patients treated with contrast-guided PVI. Of note, the presented amount of contrast agent also included the contrast agent given for PV angiography before the ablation. Additional application of contrast agent to verify complete PV occlusion by the cryoballoon was only necessary in 3/25 (12%) patients (3/100 PVs) in the OPV-group.

There were no differences between the groups regarding fluoroscopy time, cumulative radiation dose and total procedure time (Fig. 2B–D). In addition, there was no difference in left atrial (LA) dwell time (Fig. 2E), defined as the time from the transseptal puncture with the SL1 sheath until removal of all sheath and catheters from the LA. Also, no differences were observed in left atrial dwell time of the POLARSHEATH (Fig. 2F) between the groups (SL1 sheath was exchanged for POLARSHEATH after PV angiography and before preparing the POLARx cryoballoon catheter). All procedures were performed without any complications.

### Acute ablation results

In our cohort of 50 patients, a total of 200 PVs were identified and ablated (25 patients and 100 PVs per group). Patients with a left common PV ostium were excluded from the analysis in advance. All PVs (100%) were successfully isolated in procedures either contrast or pressure guided (Table 2). The number of PVs that were isolated with the first freeze cycle did not differ between the groups. Furthermore, similar results could be observed for the PVs isolated with the second freeze cycle and those that needed three or more freeze cycles.

**Table 2:** Comparison of PVs being isolated with the first, second, third or more freeze cycle between the contrast-guided and the pressure-guided procedure

	Contrast guided (*n* = 25)					Pressure guided (*n* = 25)				*p* value
	LSPV	LIPV	RIPV	RSPV		LSPV	LIPV	RIPV	RSPV	
Isolation of PV (%)	100	100	100	100		100	100	100	100	1.000
Isolation with 1 st freeze (%)	20/25 (80)	23/25 (92)	20/25 (80)	17/25 (68)		17/25 (68)	22/25 (88)	17/25 (68)	18/25 (72)	0.401
Isolation with 2nd freeze (%)	4/25 (16)	2/25 (8)	3/25 (12)	6/25 (24)		8/25 (32)	3/25 (12)	6/25 (24)	3/25 (12)	0.573
Isolation with 3rd freeze or more (%)	1/25 (4)	0/25 (0)	2/25 (8)	2/25 (8)		0/25 (0)	0/25 (0)	2/25 (8)	4/25 (16)	0.999

No significant differences between the groups were observed

### Acute ablation results per individual PV

Ablation data for each individual PV are summarized in Table 3. No significant differences regarding mean minimal temp. (°C), total freezing cycles, total freezing time, Time to PVI (s), time to − 30 °C, thawing time to 20 °C and the rate of TTI recordings were observed between the contrast-guided and the pressure-guided group for most of the PVs. A significantly longer time to − 30 °C was observed for the pressure-guided group in the LSPV (Table 3, *p* < 0.05). Further, a significantly longer thawing time to 20 °C was observed for the RSPV (Table 3, *p* < 0.05) with unclear relevance.

**Table 3:** Characteristics of freeze cycles displayed for each PV in the contrast-guided and the pressure-guided groups

Variable	Contrast guided	Pressure guided	*p* value
LSPV			
Mean minimal temp. (°C)	− 58.3 ± 6.3	− 57.0 ± 5.1	0.434
Total freezing cycles	1.2 ± 0.5	1.3 ± 0.5	0.574
Total freezing time (s)	219.7 ± 99.6	237.6 ± 87.4	0.503
Time to PVI (s)	40.5 ± 16.4	53.2 ± 24.0	0.090
Time to − 30 °C (s)	26.1 ± 2.7	28.3 ± 3.0	0.011*
Thawing time to 20 °C	52.1 ± 17.4	60.8 ± 18.3	0.119
Rate of TTI recordings (%)	17/25 (68)	14/25 (56)	0.561
LIPV			
Mean minimal temp. (°C)	− 55.6 ± 6.1	− 54.2 ± 4.5	0.359
Total freezing cycles	1.1 ± 0.3	1.1 ± 0.3	0.646
Total freezing time (s)	198.7 ± 59.5	206.4 ± 67.3	0.671
Time to PVI (s)	45.6 ± 34.8	39.2 ± 34.8	0.623
Time to − 30 °C (s)	26.1 ± 2.0	27.2 ± 2.6	0.112
Thawing time to 20 °C	44.0 ± 18.2	50.3 ± 14.9	0.198
Rate of TTI recordings (%)	18/25 (72)	12/25 (48)	0.148
RIPV			
Mean minimal temp. (°C)	− 54.9 ± 5.3	− 54.9 ± 6.2	0.980
Total freezing cycles	1.4 ± 0.9	1.6 ± 1.4	0.485
Total freezing time (s)	237.4 ± 120.4	293.2 ± 275.9	0.359
Time to PVI (s)	59.25 ± 34.2	60.7 ± 35.6	0.935
Time to − 30 °C (s)	27.4 ± 2.3	27.8 ± 2.2	0.553
Thawing time to 20 °C	46.5 ± 16.0	57.6 ± 28.4	0.128
Rate of TTI recordings (%)	8/25 (32)	9/25 (36)	0.999
RSPV			
Mean minimal temp. (°C)	− 54.2 ± 9.1	− 55.2 ± 6.3	0.667
Total freezing cycles	1.4 ± 0.8	1.5 ± 1.0	0.747
Total freezing time (s)	243.0 ± 112.5	266.0 ± 175.7	0.584
Time to PVI (s)	29.5 ± 9.5	49.5 ± 27.9	0.068
Time to − 30 °C (s)	26.5 ± 2.7	26.2 ± 1.8	0.663
Thawing time to 20 °C	38.8 ± 18.7	53.3 ± 19.0	0.018*
Rate of TTI recordings (%)	8/25 (32)	9/25 (36)	0.999

No differences were observed between the groups, except for “Time to − 30 °C” during ablation of the LSPV and “Thawing time to 20 °C” during ablation of the RSPV with unclear importance

* *p* < 0.05

## Discussion

With the present study, we provide early observational data assessing the feasibility and procedural efficacy of cryoballoon PV ablation using the novel occlusion pressure visualization tool of the SMARTFREEZE® console during cryoballoon PVI with the POLARx cryoballoon. Key findings of our analysis are:

1. The use of pressure waveform recording to confirm complete PV occlusion during cryoballoon PVI is feasible and easy to use.
2. The acute efficacy of cryoballoon PVI guided by pressure waveforms is comparable to those procedures guided by contrast injection as 100% of PVs were acutely isolated and the rate of first pass isolations as well the number of freeze cycles was comparable between the groups.
3. Pressure-guided cryoballoon PVI significantly reduced the amount of contrast agent used during the procedure. In combination with preprocedural imaging, even cryoballoon PVI procedures free of contrast agent free are possible.

The concept of pressure-waveform visualization has first been described by Siklódy et al. [12]. In their study, the authors initially advanced the cryoballoon to the PV ostium until a change in pressure waveform to a pulmonary artery pressure could be observed. Complete occlusion of the vein was then verified by transoesophageal echocardiography showing an absence of flow within the respective PV. Later Kosmidou and colleagues evaluated the pressure waveform visualization workflow in a series of 35 patients [11]. In the first 10 procedures, PV occlusion was double checked by contrast injection and pressure waveform analysis with comparable prediction of complete PV occlusion. Similarly, Raizada et al. administered contrast agent for demonstration of PV occlusion only after pressure waveform analysis made this likely, with good procedural results [18]. Hasegawa and colleagues found 91.7% of the PVs to be occluded after confirmation with contrast that was guided by pressure waveform analysis before [19]. In contrast to that, another trial found only 64% of the PV that seemed to be occluded after a respective pressure waveform analysis to be completely occluded after additional angiography [20]. However, it has to be noted that the pressure waveforms of an incomplete occlusion and a complete occlusion are sometimes not easy to differentiate as seen in the example tracing in the publication of Kosmidou [11]. It is important to note, that a complete occlusion is only present when the a-wave has completely disappeared. Our study shows that incorporation of the pressure measurement into the console leads to reproducible results and significantly reduces the contrast agent use in these procedures.

Moreover, the approach has also been combined with other techniques like intracardiac echocardiography (ICE) were PV occlusion was confirmed by absence of colour-flow Doppler signals around the periphery of the cryoballoon in addition to pressure waveform analysis [21, 22]. Alyesh et al. also introduced a workflow without fluoroscopy by combining the techniques [22].

It has to be noted, that pressure transducers and pressure visualization are not always routinely used in EP-labs which makes the use of pressure waveform analysis elaborately.

Although the concept of using pressure waveform recordings to verify PV occlusion was demonstrated before, the integration into the SMARTFREEZE® console now allows easy incorporation into the workflow during cryoballoon PVI procedures.

### Feasibility

By now, the pressure-guided approach has been presented by different groups with various modifications such as a combined use of contrast agent or in combination with TEE or ICE [11, 12, 18, 20]. However, some authors also presented procedural outcomes of procedures that were only guided by pressure guidance without the additional use of contrast or echocardiography [19, 23].

In our study, we utilized an approach using only pressure-waveform visualization in the pressure-guided group. Here, we compared the first 25 patients treated with cryoballoon PVI and the novel pressure-waveform visualization tool of the SMARTFREEZE® console with 25 patients that were ablated with our approved conventional approach. As we found no differences in procedural duration or LA dwell time of the sheaths and catheters we can postulate, that the use of the tool is not associated with an additional effort and therefore easy and reliable to use.

### Acute procedural outcomes

The most important observation made from our study is, that the acute procedural endpoint of PV isolation was achieved in both groups. It is known, that the isolation of the PVs during cryoballoon PVI is highly dependent on the complete occlusion of the vein with the balloon [24]. In our study acute isolation of all PVs were achieved in all cases. We did not observe any differences in the proportion of PVs isolated with the first or second freeze cycle between our conventional and the pressure-guided approach. It can thus be stated that both techniques are similarly suited to effectively guide the cryoballoon procedure. This goes in line with the findings of Hasegawa et al. who demonstrated that the positive predictive value of the occlusion obtained from contrast or pressure guidance to predict acute successful isolation did not differ between the approaches [19].

### Ablation characteristics

It is known that different characteristics of the freeze cycle during cryoballoon PVI like the minimal temperature of the cryoballoon during the freeze cycle, the time to isolation and the slope of rewarming are predictive of acute and chronic procedural outcome [10, 25]. Therefore, we focused our analysis in these ablation characteristics more detailed for every PV. In our study, these parameters did not significantly differ between the contrast-guided and the pressure-guided group for each respective PV, although there was a trend to a longer time to isolation in the pressure-guided group. A shorter time to isolation in the contrast-guided group could be linked to a greater push against the PV ostium in the contrast-guided group. However, minimal temperature, as well as thawing time did not differ between the groups, or the results trended into the other direction (thawing time) so that we do not interpret this to be in a causal relationship. Similarly, another recent study investigating the novel occlusion pressure tool similarly observed no differences in ablation characteristics [26]. Furthermore, here no differences in outcomes after a 6-month follow-up could be observed.

Of note, our observations are limited to cryoballoon procedures with the POLARx cryoballoon system. As mentioned above, minimal temperature and thawing time indicate long-term durable PV isolation. Here, we did not observe differences between the groups which could explain the similar long-term results presented by Sciacca et al.

It has to be mentioned that the characteristics of the freeze cycles and the temperatures observed during ablation are different from those procedure performed with other cryoablation systems [13, 15]. When compared to larger trials using the POLARx cryoballoon system we found similar ablation characteristics like minimal balloon temperature, time to isolation, total freeze cycles or thawing time [13, 15, 17].

### Advantages

Pressure waveform analysis to predict total PV occlusion during cryoballoon PVI has one obvious advantage, which is the significant reduction of the amount of contrast agent compared to the conventional approach. This might be of particular interest when treating patients with renal disease or subclinical hyperthyroidism. The reduction of contrast agent can be described as the one major advantage of all studies investigating the pressure waveform analysis approach [11, 18, 19]. Some of the studies even reported procedures without the use of any contrast agent [21, 22], which appears feasible when combining the procedure to additional imaging revealing the PV anatomy.

In addition, other trials reported a significant reduction in fluoroscopy used during the procedure when using pressure guidance [11, 19, 22]. Of note, some of these combined the pressure waveform analysis with imaging techniques like TEE or ICE [11, 21, 22]. Yet other studies reported less fluoroscopy use during the procedure without using additional imaging like we did in our study [18, 19]. Nevertheless, it is important to note, that the pressure waveform analysis approach does not increase fluoroscopy times and doses, which is important for both patients and operators.

## Limitations

As the pressure visualization tool for the SMARTFREEZE® console was only introduced recently, we can only report on a limited number of patients. For similar reasons, we are not able to provide data on clinical follow-up currently.

It would have been interesting to directly compare pressure-guided PV occlusion with contrast-guided occlusion in the same PV. In an optimal scientific approach, the techniques could have been compared in a blinded manner. However, due to the viscosity of the contrast agent pressure waveforms were attenuated after contrast injection and thereby hard to interpret.

As this study was designed only to compare intraprocedural ablation characteristics we cannot provide results on long-term efficacy.

## Conclusion

Our study with focus on cryoablation characteristics investigated the feasibility and the procedural efficacy of CB-PVI guided by the novel occlusion pressure visualization tool of the Boston Scientific SMARTFREEZE® console. Pressure-guided cryoballoon PVI showed similar intraprocedural outcomes regarding efficacy, procedural duration, LA dwell time, and radiation exposure. Confirmation of total PV occlusion by pressure guidance resulted in a significant reduction in the amount of contrast agent used. Therefore, pressure-guided cryoballoon PVI constitutes a feasible alternative to conventional contrast injection to confirm total PV occlusion.

## Data Availability

Data available on request from the authors.
